# 

**Published:** 1885-04

**Authors:** 


### BOOKS AND PAMPHLETS RECEIVED.

Contagious Diseases of Domesticated Animals. Investigations by Department of Agriculture. 1884–1885.

The Osteology of Amia Calva, including Certain References to the Skeleton of Teleosteans. By Dr. R. W. Shufeldt, U. S. Army.

Report on Cattle, Sheep, and Swine, Supplementary to Enumeration of Live Stock on Farms in 1880. Clarence Gordon, Special Agent in charge. Washington, 1884.

Transactions of the Wisconsin State Agricultural Society, Horticultural, Dairymen's Association, and Department of Agriculture of the University. Madison, Wis., 1884.

A Correction of Certain Statements Published in the Zoöphilist, and also a Castigation and an Appeal. By H. Newell Martin, M.A., M.D., D.Sc. Baltimore, 1885.

Report of Committee on School Hygiene in Tennessee. By Daniel F. Wright, M.D., Clarksville, Tenn.

Annual Report of the Board of Managers of the New York State Reformatory at Elmira, 1884.

Typhoid Fever and Low Water in Wells. By Henry B. Baker, M.D., Lansing, Mich., 1885.

Address in Medicine delivered before the Medical Society of the State of Pennsylvania. By W. H. Daly, M.D.

Should Experiments on Animals be Restricted or Abolished?

Methods of Studying the Physiological Action of Drugs.

The Absorption of Sugar and Albuminoids by the Stomach.

The Thermic Phenomena in Contraction of Mammalian Muscles. By Robert Meade Smith, Professor of Comparative Physiology in the University of Pennsylvania.

Studies from the Biological Laboratory, Johns Hopkins University. Vol. III., No. 2. Baltimore.

Proceedings of the Newport Natural History Society, 1883–4.

Johns Hopkins University Circulars.

Annals et Bulletin de la Société de Medicine de Gand.

Journals.—The Veterinarian, London; The Veterinary Journal, London; The Quarterly Journal of Veterinary Science in India; American Veterinary Review, New York; Der Zoologische Garten, Frankfort; Schweizer Archiv fur Thierheilkunde, Zurich; Archiv fur Wissenschaftliche und Praktische Thierheilkunde, Berlin; Wochenschrift fur Thierheilkunde und Viehzucht, Augsburg; Tidskrift for Veterinar-Medicin och Husdjursskotsel, Stockholm; Giornale di Anatomia, Fisiologia e Patologia, degli Animali, Pisa; Il Medico Veterinario, Torino; La Clinica Veterinaria, Milano; La Cronica Medica, Valencia; Journal de la Société contre L'Abus du Tabac, Paris; Kansas City Review of Science and Industry; The College and Clinical Record, Philadelphia; New England Medical Monthly, Sandy Hook; Nashville Journal of Medicine and Surgery; The Southern Clinic, Richmond; The Western Medical Reporter, Chicago; Journal of Cutaneous and Venereal Diseases, New York; Denver Medical Times; The St. Joseph Medical Herald; The Weekly Medical Review, St. Louis; The Polyclinic, Philadelphia; Medical World, Philadelphia; Atlantic Journal of Medicine, Richmond; Texas Courier Record, Fort Worth; The American Journal of Ophthalmology, St. Louis; Pacific Medical and Surgical Journal and Western Lancet, San Francisco; New York Medical Journal; Health and Home, Washington; American Psychological Journal, Philadelphia; Leonard's Illustrated Medical Journal, Detroit; Annals of Surgery, St. Louis; Kansas City Medical Index; Eastern Medical Journal, Worcester; The Therapeutic Gazette, a Monthly Journal, Detroit; Horseshoer and Hardware Journal, Chicago; The Blacksmith and Wheelwright, New York; National Live Stock Journal, Chicago; The Breeder's Gazette, Chicago; Cultivator and Country Gentleman, Albany; Weekly Drover's Journal, Chicago; The Chicago Tribune; Indiana Farmer, Indianapolis; Jersey Bulletin, Indianapolis; The Guernsey Breeders' Journal, Westchester, Pa.; Colorado Farmer, Denver.

